# Effects of Intra-Uterine Fluid Accumulation after Artificial Insemination on Luteal Function in Mares

**DOI:** 10.3390/ani13010067

**Published:** 2022-12-23

**Authors:** Francesca Freccero, Beatrice Mislei, Diego Bucci, Francesco Dondi, Gaetano Mari

**Affiliations:** 1 DIMEVET, Department of Veterinary Medical Sciences, University of Bologna, Via Tolara di Sopra 50, 40064 Ozzano dell’Emilia, Italy; 2 AUB INFA National Institute of Artificial Insemination, University of Bologna, Via Gandolfi 16, 40057 Cadriano, Italy

**Keywords:** corpus luteum, blood flow, progesterone, intra-uterine fluid accumulation, mares

## Abstract

**Simple Summary:**

The aim of the study was to investigate whether intra-uterine fluid accumulation after artificial insemination alters blood flow in the corpus luteum and plasma progesterone concentrations in mares. The presence of fluid was detected by ultrasound 12 h after insemination with frozen semen in 40 Standardbred mares during 53 estrous cycles in a commercial setting: luteal blood flow was measured by Power Doppler ultrasonography 3 and 6 days after ovulation, progesterone concentration was measured in peripheral plasma 6 days after ovulation, and pregnancy was diagnosed by ultrasonography 14 days after ovulation. Luteal blood flow did not differ significantly between cases with (n = 28) and without (n = 25) intra-uterine fluid accumulation after insemination, but progesterone concentrations were higher in cases of intra-uterine fluid accumulation than in cases without (9.3 ± 1.1 vs. 6.6 ± 0.5 ng/mL, *p* = 0.048). Pregnancy was less likely in cases with intra-uterine fluid accumulation than in cases without (10/28 vs. 17/25). These data suggest that although intra-uterine fluid accumulation increases the secretion of progesterone, pregnancy is more dependent on uterine health than ovarian function.

**Abstract:**

After breeding or artificial insemination, especially with frozen/thawed semen, mares often develop a persistent uterine inflammation, which is diagnosed by intra-uterine fluid accumulation. Here, we explored whether intra-uterine fluid accumulation affects corpus luteum function and tested the hypothesis that intra-uterine fluid accumulation after artificial insemination alters blood flow in the corpus luteum and plasma progesterone concentrations. A total of 40 Standardbred mares were artificially inseminated with frozen-thawed semen 30 to 36 h after induction of ovulation, and cases with or without intra-uterine fluid accumulation were detected by ultrasound 12 h after insemination. Luteal blood flow was measured by Power Doppler ultrasonography 3 and 6 days after ovulation, progesterone concentration was measured in peripheral plasma by ELISA 6 days after ovulation, and pregnancy was diagnosed by ultrasonography 14 days after ovulation. Luteal blood flow increased between 3 and 6 days after ovulation, but blood flow did not differ significantly between cases with (n = 28) and without (n = 25) intra-uterine fluid accumulation after insemination. Surprisingly, progesterone concentrations were higher in cases of intra-uterine fluid accumulation than cases without (9.3 ± 1.1 vs. 6.6 ± 0.5 ng/mL, *p* = 0.048). Pregnancy was less likely in cases with intra-uterine fluid accumulation than in cases without (10/28 vs. 17/25, *p* = 0.019), and there was a negative correlation between the severity of intra-uterine fluid accumulation and per cycle pregnancy rate. These data suggest that although intra-uterine fluid accumulation increases the secretion of progesterone, pregnancy is more dependent on uterine health than ovarian function.

## 1. Introduction

Mares show an acute inflammatory response of the uterus after mating or artificial insemination (AI) [1,2], which is part of the physiological response for effective removal of excess sperm and contaminating bacteria introduced into the uterus [3,4,5]; this inflammatory response is particularly evident in mares bred with frozen/thawed semen, regardless of the site of deposition [2,6]. Such inflammation is characterized, among other factors, by the influx of granulocytes such as neutrophils (PMN) [1] and intra-uterine fluid accumulation (IUFA) [2,7,8,9,10]. In resistant mares, mating-induced endometritis (MIE) subsides within 48 h, whereas the inflammation persists in mares susceptible to endometritis [11,12,13].

Persistent mating-induced endometritis (PMIE) is the most common cause of reproductive failure in mares [14,15], particularly in older mares and in “old maiden mares” bred for the first time after 10–12 years of age. In one study, 16% of the naturally mated mares showed persistent post-breeding IUFA [16], compared with a 30% rate for mares inseminated with frozen semen reported in another study [17].

Resistance vs. susceptibility to endometritis is a concept introduced by Hughes and Loy [11]. Delayed uterine clearance is an important etiological factor in the development of susceptibility to endometritis, and it is characterized by IUFA, which can be easily detected by ultrasonography [2,18]. The presence of IUFA has been associated with the severity of endometrial histopathology, indicating that biopsy score and ability to clear inflammation are a convincing set of criteria for identifying problem mares, and IUFA has frequently been used as a clinical tool to classify mares resistant or susceptible to PMIE [19]. Therefore, in this manuscript, IUFA detected by ultrasonography and susceptibility to PMIE are referred to the same condition.

In a previous study, Bollwein et al. (2003) [20] observed that blood flow of the ovarian artery ipsilateral to the dominant follicle was increased after induction of a uterine inflammation by intra-uterine deposition of raw semen, but the direct effects on luteal perfusion were not investigated [18]. More recently, Lüttgenau et al. (2021) [21], of the same research group, showed that an intrauterine infusion of killed deep-frozen semen increases uterine blood flow resistance and alters gene expression of inflammatory cytokines for at least 7 d, but does not affect ovarian blood supply and luteal function in mares susceptible to PMIE [21].

Here, we tested the hypothesis that IUFA after AI alters blood flow in the corpus luteum and the plasma progesterone concentrations. To test this hypothesis, we measured luteal blood flow and peripheral plasma progesterone concentrations after AI with frozen-thawed semen and compared cases with and without IUFA after breeding. In addition, the incidence of IUFA and its effect on per cycle pregnancy rate were explored.

## 2. Materials and Methods

Data were collected during two consecutive breeding seasons at the AUB National Institute of Artificial Insemination of the University of Bologna, Italy. Procedures used in this investigation were approved by the Committee for Animal Welfare, Bologna University (ref. n.1261).

### 2.1. Mares

A total of 40 Standardbred mares, (10 maiden,16 foaling, 14 barren), aged 11.4 ± 5.1 years (mean ± SD; range 5–19), weighing 470–550 kg, BCS 2.5–3/5, with parity ranging from 2 to 6 for foaling and barren mares, were bred in a commercial setting using the frozen semen of 7 stallions, employing a total of 53 estrous cycles in the study. The influence of the mare’s age on fertility was evaluated by dividing the mares into three groups (≤10, 10–15, >15 years).

Frozen semen from seven stallion was used; all ejaculates were of proven fertility based on the results obtained in the previous breeding seasons: per cycle and seasonal pregnancy rates were 38% (min–max 30% to 46%) and 68% (min–max 60% to 76%), respectively, for all stallions in a book of 10 or more mares. Semen was provided by different centers in Europe and the US, sperm concentration ranged from 150–200 × 10^6^/mL, and number of straws used per insemination was in accordance with the recommendations of the freezing center, from 1 (0.5 mL) to 8 (4 mL).

Before AI, all mares routinely underwent a breeding soundness examination, including vaginoscopy, clinical and ultrasonographic examination of the genital tract, and endometrial cytology and bacteriology evaluations. Mares were monitored by transrectal palpation and ultrasonography of the genital tract until estrous detection, and when a follicle of 30–35 mm in diameter was detected in the presence of endometrial edema, a relaxed cervix, and a positive response to a teaser stallion, ovulation was induced by the administration of 2500 IU of hCG i.v. (Vetecor, Farma Mediterrania, San Just Desvern, Spain). All mares were monitored every 4–8 h, starting 24 h after hCG administration, if ovulation had already occurred 24 h after hCG mares were not inseminated. AI were performed before ovulation, 30–36 h after hCG administration, deep in the uterine horn near the utero-tubal junction ipsilateral to the preovulatory follicle, with a 75 cm flexible, smooth-tipped pipette (Minitube, Germany, ref. 17207/1275) that was guided by manipulation per rectum, and a flexible stylet (Minitube, Germany, ref. 17209/1075). At the time of AI, all mares received 50 mg of dexamethasone i.v. (Desashock^TM^, Forte, Dodge Animal Health s.*p*.a., Europe). Ovulation (Day 0) was ultrasonographically detected between 10 and 12 h after AI; mares that failed to ovulate were inseminated again within 12 h.

A case was defined as an insemination with a confirmed ovulation within 24 h post AI; 53 cases were included in the study. At the time of ultrasonographic assessment of ovulation, cases were divided in two groups based on ultrasonographic detection of IUFA: no fluid or fluid < 2 cm depth (NF), fluid > 2 cm depth (F). Further, fluid was scored in four groups, based on the amount and/or ultrasonographic features: GRADE I, anechoic fluid > 2 cm depth in one uterine compartment (left uterine horn, right uterine horn, uterine body); GRADE II, anechoic fluid > 2 cm depth in two or more uterine compartments; GRADE III, fluid > 2 cm depth with hyperechoic spots; and GRADE IV, hyperechoic fluid > 2 cm depth.

NF cases received 20 IU of oxytocin i.m. (Izossitocina, Izo s.r.l., Brescia, Italy) and F cases received a uterine lavage with 2 L of pre-warmed Ringer lactate solution, followed by 20 IU of oxytocin i.m.

B-mode and Power Doppler (PD) ultrasound assessments of the CL were performed on Days 3 and 6, and blood samples for P4 determination were harvested on Day 6 after ovulation, as described below. Pregnancy diagnosis was performed 14 days after ovulation by transrectal ultrasonography.

### 2.2. Ultrasonographic Assessment of Luteal Blood Flow

**Figure 1:**
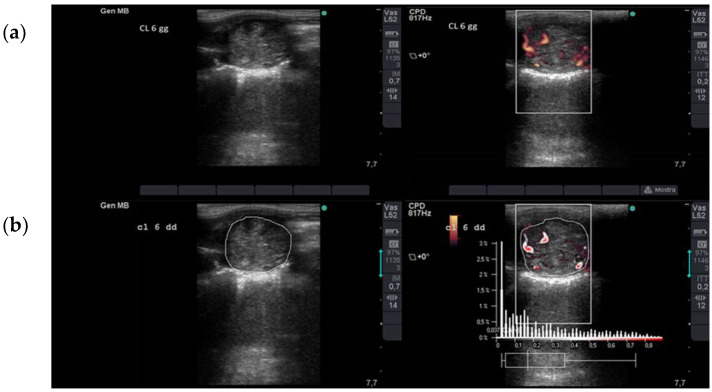
Ultrasonographic assessment of luteal blood flow. (**a**) B-mode (**left**) and Power Doppler (**right**) images of a corpus luteum on Day 6 after ovulation. The images are acquired at the maximum cross-sectional area of the corpus luteum. Using the Power Doppler mode, the color box is superimposed to the corpus luteum, and the blood-flow within the vessels is depicted as colored pixels. (**b**) Computer-assisted image analysis for semi-quantitative estimation of luteal blood flow. The corpus luteum cross-sectional area and the colored pixel area were measured using the software caliper from the B-mode (**left**) and Power Doppler (**right**) images, respectively.

B-mode and Power Doppler ultrasonographic assessments of the CL were performed on Days 3 and 6 after ovulation in each estrous cycle. Time points were chosen based on the results of previous studies [22,23,24]. Briefly, mares were restrained in stocks without sedation nor other physical restraint, and the examination was performed by the same operator using a linear 5–10 MHz transrectal probe and an ultrasound machine (Sonosite MicroMaxx; Fujifilm Sonosite, WA, USA). The ultrasound setting for depth, Doppler gain, pulse repetition frequency, and wall filter were identical for each examination. The CL was first identified in B-mode, and images were acquired at the maximum cross-sectional area; then, using the same view, the Power Doppler mode was activated to record the blood flow in the sample box over imposed to the CL. Images with maximum numbers of colored pixels were acquired; at least three images for each modality were acquired (Figure 1) and stored for off-line analysis.

The CL images were evaluated using computer-assisted image analysis software (PixelFlux™, Version 1.0; Chamelon Software, Leipzig, Germany) for the semi-quantitative estimation of LBF, expressed as the proportion of the CL area. The CL cross-sectional area and the colored pixel area were measured using the software caliper from the B-mode and PD-mode images, respectively. Three measurements for each modality were averaged to calculate the ratio (colored pixel area/cross-sectional area, arbitrary units AU%).

### 2.3. Blood Sample and Progesterone Assay

Blood samples were collected from the jugular vein on Days 3 and 6 after ovulation using a 18G needle into heparinized vacuum tubes. Samples were centrifuged at 600× *g* for 10 min and stored at −20°C until they were analyzed for progesterone. Progesterone was measured by the Clinical Pathology Laboratory of the University of Bologna, using an automated analyzer (Progesterone Immulite^®^ and Immulite 2000 analyzer, Siemens Healthcare s.r.l., Milan, Italy) [25]. Quality control was performed using standards (Liquicheck Immunoassay Plus Control, Biorad), as recommended by the manufacturer; the intra- and inter-assay coefficients of variation were < 10 and < 11%, respectively, and the limit of detection of the assay was 0.2 ng/mL.

### 2.4. Statistical Analysis

The statistical unit was defined as each case evaluated. Data were analyzed using R Version 3.5.2. (https://www.r-project.org/, accessed on 19 December 2018) and SPSS Version 26 (IBM Corp, Armonk, NY, USA), and significance was ascribed when *p* < 0.05. Data were evaluated for normal distribution using the Shapiro–Wilk test. The effects on luteal blood flow and progesterone concentrations were analyzed using ANOVA, fitting horse, cycle number, and age, as well as IUFA, in the model. The effects of semen volume and number of inseminations on endometritis grade were analyzed using generalized linear models. The frequency of cases that resulted in pregnancy was analyzed using Pearson’s chi-squared test. Correlations between the IUFA grade and other variables were analyzed using Spearman’s rank correlation coefficient.

## 3. Results

### 3.1. IUFA

**Figure 2:**
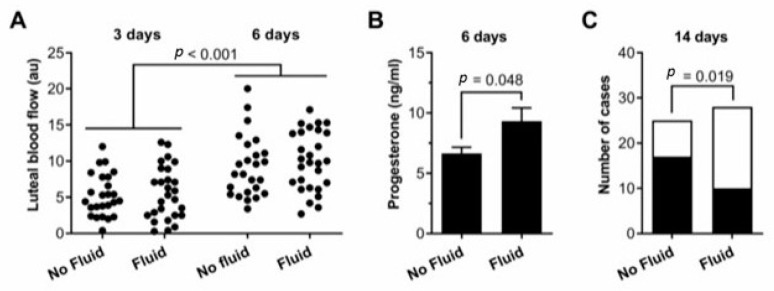
IUFA increase progesterone and reduces per cycle pregnancy rates. (**A**) Dot plot of blood flow 3 and 6 days after ovulation, in cases with IUFA or not, after insemination, with each dot representing an independent case (n = 53). Data were analyzed by ANOVA, and *p* values were reported. (**B**) Plasma progesterone concentrations 6 days after ovulation in cases with IUFA (n = 28) or without IUFA after insemination (n = 25). Data are presented as mean (SEM), and were analyzed by independent *t*-test. (**C**) Frequency of cases that resulted in pregnancy (■) or not (□), with data compared using Pearson’s Chi-squared test. IUFA: intrauterine fluid accumulation.

IUFA, as determined by the presence of > 2 cm depth of intrauterine fluid after insemination, was detected in 28 cases, while 25 cases showed no fluid, or fluid < 2 cm depth. Luteal blood flow increased between 3 and 6 days after ovulation (ANOVA, *p* < 0.001, Table 1). However, luteal blood flow measured 3 days or 6 days after ovulation did not differ between cases with IUFA or not after insemination (*p* = 0.45, Figure 2A, Table 1), whereas the peripheral plasma progesterone concentrations were higher in cases with than without IUFA after AI (Figure 2B, Table 1). Furthermore, pregnancy was less likely in cases with than without IUFA (14 mares (36%) vs. 26 mares (68%), Figure 2C).

### 3.2. Per Cycle Pregnancy Rate–Pregnancy

**Figure 3:**
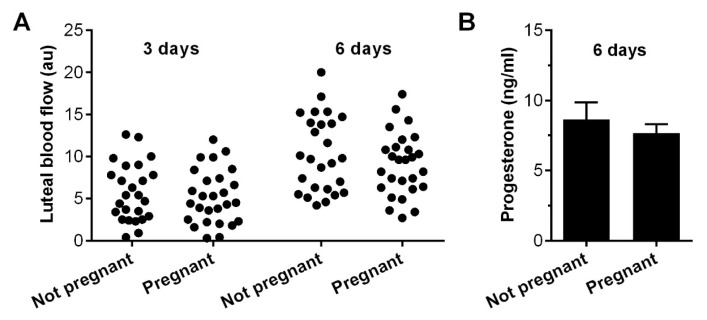
Luteal blood flow, progesterone concentrations, and per cycle pregnancy rate. (**A**) Dot plot of blood flow 3 and 6 days after ovulation for cases that did not conceive (n = 26) compared with cases that resulted in pregnancy (n = 27). (**B**) Plasma progesterone concentrations 6 days after ovulation for cases that did not conceive (n = 26) compared with cases that resulted in pregnancy (n = 27). Data are presented as mean (SEM).

There was no significant difference in luteal blood flow 3 days (ANOVA, *p* = 0.57) or 6 days (*p* = 0.26) after ovulation for cases that did not conceive compared with cases that resulted in pregnancy (Figure 3A). Similarly, peripheral plasma progesterone concentrations did not differ significantly between cases that resulted in pregnancy or not (independent *t*-test, *p* = 0.47, Figure 3B).

### 3.3. Fluid Grade

**Figure 4:**
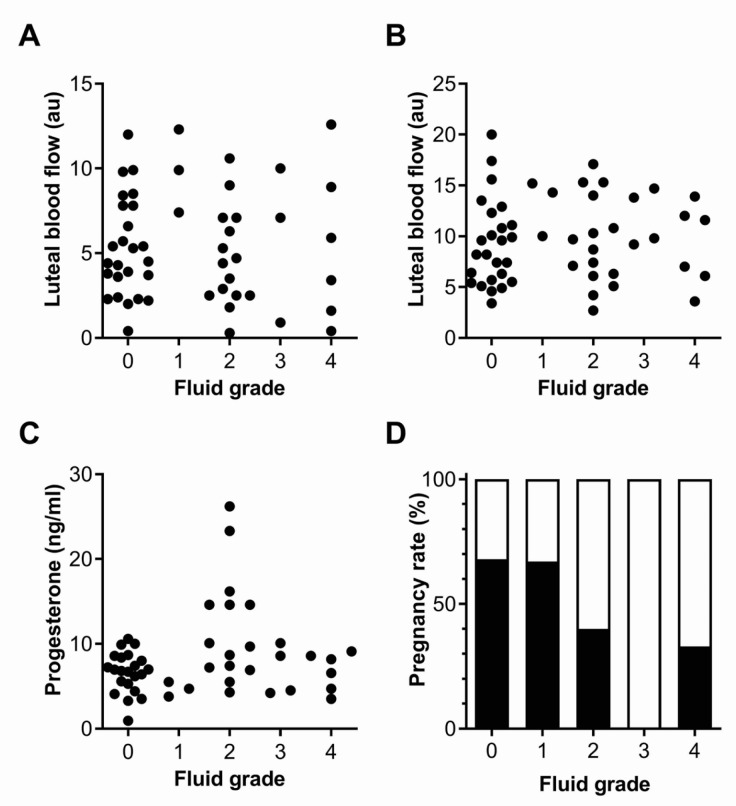
Fluid grade. (**A**–**C**) Scatterplots for luteal blood flow 3 days (**A**) and 6 days after ovulation (**B**), and for plasma progesterone concentrations 6 days after ovulation (**C**), for each fluid grade of endometritis. Grade 0 = NF. (**D**) Percent of cases by endometritis-fluid grade that did not conceive (□) or resulted in pregnancy (■).

The IUFA grade was not significantly correlated with luteal blood flow 3 days after ovulation (Spearman’s rank correlation coefficient, r = 0.02, *p* = 0.90, Figure 4A), or 6 days after ovulation (r = 0.08, *p* = 0.58, Figure 4B). Similarly, the IUFA grade was not statistically correlated with the peripheral plasma progesterone concentrations (r = 0.18, *p* = 0.23, Figure 4C). There was no significant association between the IUFA grade and age (r = 0.15, *p* = 0.29), number of inseminations per case (r = −0.20, *p* = 0.16), or the volume inseminated (r = 0.01, *p* = 0.99). However, there was a significant negative correlation between the IUFA grade and per cycle pregnancy rate, with the highest for grade 0 and 1 (r = 0.36, *p* < 0.01, Figure 4D).

### 3.4. Age of the Mares and per Cycle Pregnancy Rate

No significant difference was found between the age of the mares and the per cycle pregnancy rate (Chi-squared, *p* = 0.96) (Table 2).

## 4. Discussion

In the present study, in agreement with the results of previous studies [22,23], we found that luteal blood flow increased between 3 and 6 days after ovulation, and did not differ significantly between cases with or without IUFA, AI, or by the induction of pregnancy. Plasma progesterone concentrations 6 days after ovulation were similar to those previously reported [22,23], and were higher in cases with IUFA compared to without IUFA.

This data is in disagreement with the results from only one previous study [21], in which no differences were found in plasma P4 concentrations 6 days after ovulation between mares resistant and susceptible to PMIE. It is worth noting that in that study, performed in controlled experimental conditions using 10 mares, killed-deep frozen semen was infused into the uterine body, while in our study, frozen-thawed live sperm were inseminated deep into the utero-tubal junction in a higher number of mares.

A mechanism that may help explain this observation is that inflammatory mediators, such as nitric oxide (NO) and cytokines, stimulate the in vitro secretion of progesterone and angiogenesis in the corpus luteum, playing a role in luteal development and function [26,27]. In fact, susceptible mares have an impaired ability to evacuate the uterine fluid after breeding [13,28]. This compromised uterine contractility is mediated by increased endometrial production of NO, a potent muscle relaxant found to be elevated after breeding in mares susceptible to endometritis when compared to the levels in resistant mares [13,28,29].

A potential limitation of a study in a commercial setting is that the AI protocols for frozen/thawed semen include different treatments to reduce uterine inflammation, inducing immunomodulation, supporting the uterine clearance mechanism, and optimizing pregnancy rates [30,31,32,33] because susceptible mares can be difficult to identify prior to breeding, as most of them do not show a positive uterine culture at this time [21], and clinical signs, such as ultrasonographic and laboratory findings, vary between pathogens [34]. With this assumption, it is noteworthy that the evaluation of any potential influence of the protocol used (i.e., dexamethasone) on the results obtained was beyond the aim of our study and would need to be investigated in a different study setting. However, whether or not an influence from the treatment on the results can be ruled out, it is also important to note that all the mares in both groups underwent the same protocol. In this regard, in a previous study, the same treatment (50 mg of dexamethasone i.v.) had no effect on intrauterine NO in susceptible mares inseminated with killed spermatozoa [35].

In this study, the percentage of estrous cycles with IUFA after AI was 52.8%, which is higher than that previously reported [7,8,10], and this adds further details to the observations of these studies. The reason could be that no selection was made on mares, even if in some cases (barren for more than two years, older age, poor perineal conformation, pendulous uterus), a recommendation could have been made to avoid the use of frozen semen in those mares. In fact, in the study of [7], mares with a history of low fertility were not accepted as candidates for insemination with frozen semen.

In agreement with previous studies [8,17,36], we found that the per cycle pregnancy rate was statistically lower in the group with than without IUFA (35.7% vs. 68% respectively).

In our conditions, neither the number of AI per cycle nor the volume inseminated affected presence or grade of IUFA; however, it is possible that these findings were affected by the small number of cycles included in the study and the protocol used at the time and after AI and thus, need to be confirmed.

Similarly, the age of the mare did not show any effect on PCPR; however, this result could be affected by the relatively low number of “aged” mares included. Scoggins (2005) [37], reported that advancing age adversely affects the Thoroughbred broodmare’s reproductive efficiency, and that causes for the decline in fertility include decreased oocyte and embryo quality, anatomical defects, and endometrial degeneration. In this review, mares were divided in four groups by age (2–8, 9–13, 14–18, >18), and a significant reduction in PCPR between 15–21 days, seasonal pregnancy rate at 40–42 days, and live foal rate were observed in the two groups of broodmares older than 14 years [37]. In our study, 10 out of 40 mares were older than 15 years, representing a total of 16 out of 53 estrous cycles, and this may explain why our findings are different from those in previous studies [7,8].

This study has some limitations, mainly attributed to the clinical setting, that have been acknowledged previously; notably, the fact that all the mares received a dexamethasone injection after breeding. Clinical studies are less able than intervention studies to control for various potential confounding factors, such as age, cycle number, sperm quality, treatments, or disease susceptibility. Notwithstanding these limitations, clinical studies are important because they often include access to more mares than intervention studies, and the mares better reflect the clinical population of animals inseminated with frozen-thawed semen.

## 5. Conclusions

In agreement with previous studies, we found that luteal blood flow did not differ significantly between cases with IUFA or not. Surprisingly, IUFA was associated with increased progesterone concentrations, and this link requires further investigations in controlled experimental studies, possibly aimed to evaluate intra-uterine levels of NO and its relationship to LBF and P4 secretion in mares resistant and susceptible to PMIE.

## Figures and Tables

**Table 1:** Differences in luteal blood flow at Days 3 and 6 and P4 levels at Day 6 in groups with and without intra-uterine fluid accumulation.

Group (Mares n °)	LBF (a.u.) 3 d	LBF (a.u.) 6 d	P4 (ng/mL) 6 d
F (22)	6.13 ± 4.58 *	10.05 ± 4.08 *	9.31 ± 5.71 °
NF (18)	5.30 ± 2.91 *	9.25 ± 4.23 *	6.64 ± 2.39 °
All (40)	5.74 ± 3.87 *	9.67 ± 4.13 *	8.11 ± 4.69

F: fluid > 2 cm mares; NF: no fluid or fluid < 2 cm mares; LBF: luteal blood flow (arbitrary units); P4: progesterone concentration; * indicates significant difference (*p* < 0.05) within the row (between Days 3 and 6); ° indicates significant difference (*p* < 0.05) within the column (between F and NF groups).

**Table 2:** Per cycle pregnancy rate in pregnant and non-pregnant mares divided into groups according to age.

Age (n)	Pregnant n (%)	Non-Pregnant n (%)
≤10 (22)	14 (53.8)	12 (46.2)
10–15 (11)	8 (50)	8 (50)
>15 (7)	6 (54.5)	5 (45.5)

Age (n): group by age (years) and number of mares in each group (n).

## Data Availability

The data presented in this study are available on request from the corresponding author.
